# Causal knowledge graph analysis identifies adverse drug effects

**DOI:** 10.1093/bioinformatics/btaf661

**Published:** 2025-12-12

**Authors:** Sumyyah Toonsi, Paul N Schofield, Robert Hoehndorf

**Affiliations:** Computer, Electrical and Mathematical Sciences & Engineering Division, King Abdullah University of Science and Technology, Thuwal 23955, Saudi Arabia; Department of Physiology, Development & Neuroscience, University of Cambridge, Cambridge, CB2 3EG, United Kingdom; Computer, Electrical and Mathematical Sciences & Engineering Division, King Abdullah University of Science and Technology, Thuwal 23955, Saudi Arabia; KAUST Center of Excellence for Generative AI, King Abdullah University of Science and Technology, 4700 King Abdullah University of Science and Technology, Thuwal, Makkah, 23955, Saudi Arabia; KAUST Center of Excellence for Smart Health (KCSH), King Abdullah University of Science and Technology, 4700 King Abdullah University of Science and Technology, Thuwal, Makkah, 23955, Saudi Arabia

## Abstract

**Motivation:**

Knowledge graphs and structural causal models have each proven valuable for organizing biomedical knowledge and estimating causal effects, but remain largely disconnected: knowledge graphs encode qualitative relationships focusing on facts and deductive reasoning without formal probabilistic semantics, while causal models lack integration with background knowledge in knowledge graphs and have no access to the deductive reasoning capabilities that knowledge graphs provide.

**Results:**

To bridge this gap, we introduce a novel formulation of Causal Knowledge Graphs (CKGs) which extend knowledge graphs with formal causal semantics, preserving their deductive capabilities while enabling principled causal inference. CKGs support deconfounding via explicitly marked causal edges and facilitate hypothesis formulation aligned with both encoded and entailed background knowledge. We constructed a Drug–Disease CKG (DD-CKG) integrating disease progression pathways, drug indications, side-effects, and hierarchical disease classification to enable automated large-scale mediation analysis. Applied to UK Biobank and MIMIC-IV cohorts, we tested whether drugs mediate effects between indications and downstream disease progression, adjusting for confounders inferred from the DD-CKG. Our approach successfully reproduced known adverse drug reactions with high precision while identifying previously undocumented significant candidate adverse effects. Further validation through side effect similarity analysis demonstrated that combining our predicted drug effects with established databases significantly improves the prediction of shared drug indications, supporting the clinical relevance of our novel findings. These results demonstrate that our methodology provides a generalizable, knowledge-driven framework for scalable causal inference.

**Availability and implementation:**

The data is available through https://github.com/bio-ontology-research-group/Mediation-Analysis-using-Causal-Knowledge-Graph.

## 1 Introduction

Many computational biomedical tasks are inherently knowledge-based; they cannot simply be “learned” from data but require combining observations with structured background knowledge (Alterovitz and Ramoni 2011). This integration between knowledge and data becomes particularly important when addressing complex problems such as drug safety monitoring, where rare but significant adverse events must be detected despite their low prevalence in general populations (Pande 2018, Hammad *et al.* 2023).

Knowledge graphs (KGs) are the main approach for organizing biomedical knowledge, and can be used to represent biomedical entities (e.g. diseases, drugs, proteins) and their relationships in a structured format (Hogan *et al.* 2022). These graphs encode qualitative knowledge—facts that are either true or false—and have been widely applied across life sciences to represent taxonomic hierarchies, molecular interactions, and clinical associations (Zhan 2024). While KGs are very useful for representing knowledge, they lack formal mechanisms to support probabilistic and causal inference necessary for many biomedical applications.

In clinical and epidemiological research, qualitative causal relations can be expressed through directed acyclic graphs (DAGs) (Tennant *et al.* 2021). These DAGs are a component of structural causal models (SCMs) which combine causal DAGs with structural equations (Pearl 2009b). SCMs can be used to distinguish causal effects from associations and to answer “why” questions, i.e. how a probability distribution will change as a result of an intervention. SCMs and KGs remain largely separate frameworks: although the qualitative parts of an SCM (the DAG) can be embedded within a KG, and KGs may encode relations that are causal or have causal implications, it remains challenging to integrate the quantitative parts of SCMs with KGs.

Drug safety monitoring is one application that requires both knowledge representation and causal inference capabilities. Post-marketing surveillance aims to identify adverse drug reactions (ADRs) that may not have been detected during clinical trials due to their rarity or delayed onset (Raj *et al.* 2019, Trifirò and Crisafulli 2022). This surveillance depends on background knowledge (disease and drug classifications and their interrelationships, known disease progression, drug indications, and known drug side effects) and observational data (frequencies of event occurrences and co-occurrences). The surveillance task also requires causal inference to determine whether an observed association represents a genuine drug effect or results from confounding factors (Pande 2018, Hammad *et al.* 2023).

Current approaches to ADR detection include feature-based predictive models using drug descriptors, and observational data analysis from adverse event reporting systems and electronic health records (Zhang *et al.* 2020, Fukuto *et al.* 2021, Hu *et al.* 2024). While these methods are very useful, they do not fully utilize available background knowledge, nor do they account for all confounding variables. Causal inference methods like mediation analysis (Imai *et al.* 2010) offer a direct methodological framework for determining potential ADRs but rely on causal models which are often hand-crafted and do not integrate with existing background knowledge (Xu *et al.* 2015, Tchetgen and Phiri 2016, Gentreau *et al.* 2023), which limits their scalability.

We have developed a theoretical framework that integrates KGs and SCMs which we call Causal Knowledge Graphs (CKGs). CKGs extend knowledge graphs by incorporating probability distributions over graph nodes and explicitly identifying relation types with causal semantics. This integration allows us to automatically identify confounding variables based on graph structure, can generate hypotheses that align with domain knowledge through KG queries, and enables probabilistic inference that respects KG semantics (in particular the hierarchical relationships between entities).

We demonstrate the utility of our approach by applying it to ADR detection using data from UK Biobank and MIMIC-IV. Our CKG-based method successfully identifies known adverse drug reactions while also discovering novel ones not previously documented. To validate these novel findings, we apply the ADRs to the task of drug repurposing, testing whether drugs with similar adverse event profiles share therapeutic indications. Our results show significant improvement over approaches using only established ADRs, confirming the value of the ADRs we discover. The theoretical CKG framework we developed combines biomedical knowledge representation and causal inference. it has applications that extend beyond pharmacovigilance to any domain where both observational data and background knowledge with causal components are available.

## 2 Materials and methods

### 2.1 Data

We obtained causal relations between diseases from a Directed Acyclic Graph (DAG) representing disease progression/sequelae (Toonsi *et al.* 2024). The content of this DAG was text-mined from the scientific literature, and filtered using several methods to retain correct disease–disease pairs as evaluated. In the DAG, a causal relationship between two diseases indicates that the causative disease can lead to the onset of the outcome disease.

For indications, we used the high-precision subset of the MEDI-C dataset (Zheng *et al.* 2021) which is based on mined data from EHR data and multiple literature resources. In MEDI-C, drugs are mapped to RxNorm identifiers (Nelson *et al.* 2011) and diseases are mapped to the International Classification of Diseases 9th and 10th versions (ICD-10, ICD-9). For side effects of drugs, we utilized the OnSIDES dataset (Tanaka *et al.* 2024), where drugs are mapped to RxNorm and diseases are mapped to MedDRA terms (Mozzicato 2009). OnSIDES was generated by text-mining structured drug labels with a fine-tuned language model that demonstrated high performance upon evaluation; we used version 2.1.0 of the dataset. Additionally, we used the OFFSIDES dataset (Tatonetti *et al.* 2012) as an additional evaluation set of side effects. OFFSIDES was created based on statistical analysis of the FDA Adverse Event Reporting System while controlling for possible confounding factors including concomitant medications, demographics, and medical history.

We utilized two large cohorts to define the probability distributions used for causal inference and mediation analysis: the UK Biobank, and the MIMIC-IV dataset.

UK Biobank (UKB) is a prospective cohort of more than half a million participants aged 40–69 years (Sudlow *et al.* 2015), and reports diagnoses of individuals using the International Classification of Diseases (ICD). Hospital inpatient data in UKB includes diagnoses before cohort enrollment began, enabling detection of pre-existing conditions. Additionally, the UKB provides extensive data from questionnaires and verbal interviews including data on medications taken by participants. Medications are assigned identifiers unique to the UKB. In addition to basic demographic and socioeconomic data, UKB provides data about smoking, alcohol intake, and physical activity of participants.

The Medical Information Mart for Intensive Care IV (MIMIC-IV) is a dataset of electronic health records (EHRs) covering 364 627 individuals. It includes hospital records of patients admitted to the Intensive Care Unit (ICU) with diagnoses available in the International Classification of Diseases 9th, and 10th versions (Steindel 2010). Data on prescribed medications is also available where medications are expressed in free text form. We obtained data on drug use from the pharmacy records. The dataset also includes data on age, sex, ethnicity, and basic measurements like Body Mass Index (BMI).

For the statistical analysis, we used longitudinal data from UKB and MIMIC-IV, extracting ICD diagnoses with dates and drug prescription information (self-reported in UKB, pharmacy records in MIMIC-IV). We automatically mapped drugs from both cohorts to RxNorm using an automated hybrid approach (see Supplementary section Mapping of medications and conditions, available as supplementary data at *Bioinformatics* online). To ensure temporal validity, we excluded individuals without follow-up data and those whose records did not follow the required order of indication → drug use → side effect. In particular, we removed cases where the outcome preceded the indication or drug, or where drug use occurred only before the indication diagnosis (see Supplementary section Sample selection, available as supplementary data at *Bioinformatics* online for further details).

### 2.2 Causal knowledge graph construction

Knowledge can be represented in structured forms that are interpretable by machines, such as knowledge graphs (KGs). A KG can be represented as a tuple


K=(V,E,R)

where *V* are nodes representing entities and E⊆V×R×V is a set of directed edges labeled by relations in *R*. KGs enable reasoning over complex interconnections in data. In contrast, Structural Causal Models (SCMs) (Pearl 2009b) offer a formal framework specifically designed to represent and reason about causal relationships between random variables. In SCMs, a directed acyclic graph (DAG) is used where each node represents a variable, and each directed edge represents a direct causal effect from one variable to another. SCMs further define functions for each variable *x* as $x=f(pa_{x},u_{x})$ where $pa_{x}$ denotes variables with outgoing edges into *x*, and $u_{x}$ represents the errors due to unobserved factors.

All edges in SCMs are interpreted as causal relationships, which is in contrast to knowledge graphs that can include multiple types of relations. Some knowledge graphs may also include subsumption relations that structure nodes into subsumption hierarchies (Pham *et al.* 2020), representing, for example, diseases and their subtypes.

We introduce the concept of a *Causal Knowledge Graph (CKG)*, which integrates the flexible structure of KGs with causal semantics and a probabilistic interpretation, while adhering to the constraints defined by KG relationships. We formally define a Causal Knowledge Graph as follows:

Definition (Causal Knowledge Graph). *Let* K=(V,E,R)  *be a knowledge graph. Let* Ω  *be a non-empty set representing the population (e.g. individuals), and let* $2^{\Omega }$  *denote the power set of* Ω.

*A* ***Causal Knowledge Graph (CKG)*** *is a tuple:*
 

$$G=(K,R_{\mathrm{causal}},\Omega ,f,P)$$

*where:*

-
$R_{\mathrm{causal}}\subseteq R$ *is the subset of relations that are interpreted as causal*,
-
$f:V\to 2^{\Omega }$ *is a function that assigns to each node a subset of the population* Ω,
-
$P:2^{\Omega }\to [0,1]$ *is a probability measure over subsets of* Ω.

*The triple* $\left(\Omega ,2^{\Omega },P\right)$  *forms a probability space over the population. Each node* v∈V  *is assigned a probability by applying P to its corresponding subset* f(v)⊆Ω*; that is*, P(f(v)).

*The function f may be subject to constraints derived from the relations in R.*

In our work, we apply a constraint to ensure that *f* respects the is_a hierarchy between diseases by imposing the condition:


∀(u,is_a,v)∈E, ∀x∈Ω, x∈f(u)→x∈f(v)

In the CKG, the probability measure *P* associates each entity with a random variable, enabling the interpretation of relationships between entities as constraints on the joint distribution over these variables. This allows us to explicitly model causal relationships between variables and apply causal inference methods to the causal subgraph, while preserving the semantics of selected non–causal relations.

**Figure 1.**
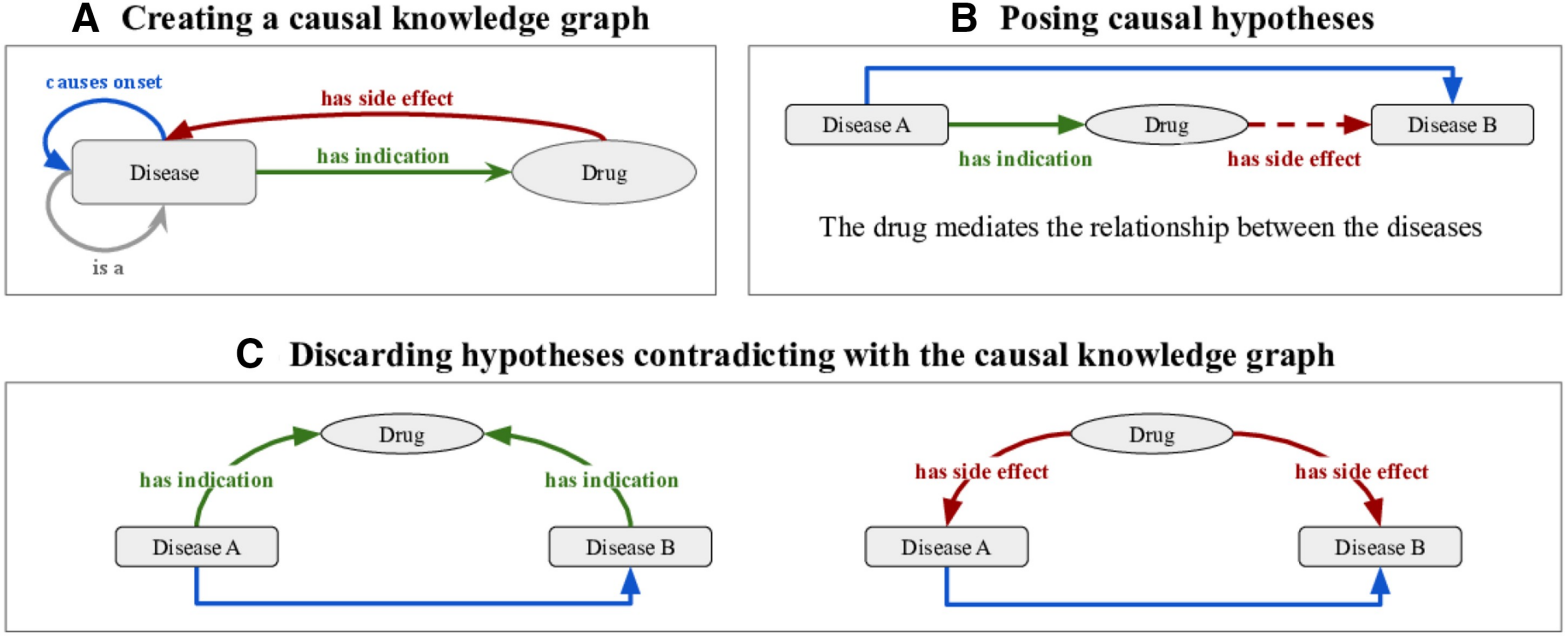
The process of generating hypotheses from the CKG.

For this study, we created a CKG with nodes representing diseases and drugs (the Drug–Disease Causal Knowledge Graph, DD-CKG). The nodes in the DD-CKG are connected by the following types of relations: disease progression, spanning 7586 edges (extracted from a DAG of causal disease relations); indications, comprising 20 955 edges (derived from MEDI-C); side effects of drugs, represented by 59 119 edges (sourced from OnSIDES); and the ICD-10 disease hierarchy, encoded as is_a relations, as illustrated in Fig. 1A. As an example, in the ICD-10 hierarchy, “Type 2 diabetes mellitus without complications” (E11.9) is_a “Type 2 diabetes mellitus” (E11). The inverse of indication edges (interpreted as a disease leading to the prescription of a drug) and disease progression edges together form the causal relation subset $R_{\mathrm{causal}}$ in the CKG. The ICD-10 hierarchy was used to impose the is_a constraint on the mapping function *f* as described in the Causal Knowledge Graph definition. The population set Ω consisted of individuals drawn from either the UKB or MIMIC-IV.

### 2.3 Generation of hypotheses

We generated candidate hypotheses of drug-mediated disease interactions using two sources of candidate disease progression or sequel relations. The first source was our CKG, from which we extracted directed pairs of the form $x\overset{\text{causes}\;\text{onset}}{\to }y$ where *x* and *y* are diseases. For each such pair, we queried the CKG for drugs indicated for the source disease *x*, forming candidate hypotheses of the form: disease *x* (indication) $\overset{\text{causes}\;\text{prescription}}{\to }$ drug $\overset{\text{causes}\;\text{onset}}{\to }$ disease *y* (potential side effect). As shown in Fig. 1B, these hypotheses represent potential cases where a drug mediates the causal relationship between diseases. As shown in Fig. 1B, these hypotheses reflect potential cases where a drug mediates the causal relationship between diseases.

The second source of hypotheses consisted of statistically significant comorbidities between diseases identified from the UKB cohort. We computed the relative risk (RR) between co-occurring diseases following the approach in Hidalgo *et al.* (2009). After correcting for multiple comparisons using the Benjamini–Hochberg procedure (α=0.05), we retained 34 843 significant associations. To orient these associations, we used diagnosis timestamps: for each disease pair (x,y), if *x* was diagnosed before *y* in the majority of cases in the UKB, we interpreted the direction as x→y.

From both sources, we constructed initial hypotheses and applied filtering to exclude hypotheses that could already be explained by knowledge encoded in the CKG as illustrated in Fig. 1C. In particular, we removed:

1. Hypotheses where a drug was indicated for *both* diseases *x* and *y*, which can reflect general treatment overlap rather than a mediating effect.
2. Hypotheses where both diseases were listed as side effects of the same drug, which would imply a common downstream effect rather than a directed causal chain.

This process can be formulated as a SPARQL query (Prud’hommeaux and Seaborne 2008) on the CKG (shown in the Supplementary section SPARQL query for contradicting hypotheses, available as supplementary data at *Bioinformatics* online).

As a result, we retained only hypotheses where the drug was indicated for the source disease, could plausibly contribute to the onset of the target disease, and there are no alternative explanations already existing in the graph. We further excluded hypotheses that lacked sufficient sample sizes, specifically cases where the drug or either disease did not appear in the data, or where no individuals had all three components of the hypothesis co-occurring (indication = 1, drug = 1, side effect = 1). This filtering process yielded 12 561 hypotheses based on causal progression pairs and 81 610 hypotheses based on comorbidity pairs.

### 2.4 Statistical analysis

Mediation analysis allows us to decompose causal effects into two components: effects that flow through an intermediate variable (the mediator) versus effects through other pathways. In our context, we test whether drugs mediate the relationship between their indications and downstream diseases. To study the mediating effects of drugs between indications and outcomes, we use causal mediation analysis grounded in the potential outcomes framework. Let *T* denote the treatment (indication), *M* the mediator (drug), and *Y* the outcome (side effect). We are interested in the *natural indirect effect (NIE)*, which quantifies the part of the effect of *T* on *Y* that operates through the mediator *M* rather than through direct paths.

Following the definition in Pearl (2014), the NIE is given by $\text{NIE}=E[Y_{0,M_{1}}-Y_{0,M_{0}}],$ where $Y_{t,M_{t^{\prime }}}$ denotes the potential outcome if treatment were set to *t* and the mediator to the value it would have had under treatment $t^{\prime }$. Potential outcomes represent the values the outcome would attain under specific interventions. This captures the change in the outcome caused by shifting the mediator from its untreated to treated value, while keeping the treatment fixed at the baseline T=0.

To estimate the NIE, we used the R package mediation (Tingley *et al.* 2014) which estimates the potential outcomes using regression models. In the mediation package, the NIE is identified as the Average Causal Mediation Effect (ACME). The ACME is identified under the sequential ignorability assumption, namely that, conditional on the observed pre-treatment covariates *X*, (i) the treatment *T* is independent of all potential mediator and outcome values, and (ii) the observed mediator *M* is independent of all potential outcomes given *T* and *X*. This assumption requires that we properly adjust for confounding variables—variables that influence both the treatment and outcome, potentially creating spurious associations. Our approach to identifying and adjusting for such confounders is described in Section 2.5. We adjusted for variables that could create such spurious associations by including them as covariates in the regression models and included interaction terms (T×M) when statistically significant (p<0.05). Estimation was performed with 1000 quasi-Bayesian simulations with heteroskedasticity-consistent standard errors. Finally, we applied multiple testing correction via the Benjamini–Hochberg procedure on the mediation results with α=0.05.

### 2.5 Confounding control

Confounding occurs when a third variable influences both the treatment (indication) and outcome (side effect), creating spurious associations that can be mistaken for causal effects (Pearl 2009a). To statisfy the assumptions of the causal mediation effect, we correct for pre-treatment confounders. Specifically, we considered sex, age, ethnicity, and BMI as potential confounders. Similar to other approaches using mediation analysis in UKB (Gentreau *et al.* 2023), we also included education, Townsend deprivation index (TDI), alcohol use, smoking, and physical activity when analyzing data in UKB; this information is not available in MIMIC-IV. To account for disease severity which in not directly observed, we used the number of comorbid diseases and the number of prescribed drugs as proxies because they can correlate with disease severity (Forslund *et al.* 2021).

We control for concomitant drug use and comorbid conditions using the information in our CKG. Specifically, we capture comorbid conditions by disease–disease causal edges, and concomitant drug exposures via indication edges. Based on the edges with relations belonging to $R_{\mathrm{causal}}$ in our CKG, we applied two graph-based criteria to identify adjustment sets: (i) the backdoor adjustment criterion which identifies confounders through backdoor paths which are paths between the treatment and the outcome that go through a common cause (a confounder), creating a spurious association in a causal graph (Pearl 2009a); and (ii) the disjunctive cause criterion which selects direct causes of the treatment, the outcome, and both (Vander Weele and Shpitser 2011). Both approaches aim to block spurious associations while preserving the causal effect of interest. For each hypothesis independently, we first added a causal edge from the drug to the potential side effect then identify confounders through applying one of the criteria. In the adjustment sets, it sometimes happened that both a disease and its more specific child (according to the ICD-10 hierarchy) appeared together. To avoid redundant adjustment, we pruned these by keeping only the parent disease and removing the child disease.

We used the DAGitty package in R (Textor *et al.* 2016) to apply the backdoor adjustment. we only considered the first 1000 adjustment sets returned by DAGitty. Among the returned sets, we selected the one with the lowest cardinality. If multiple sets shared the minimum cardinality, we retained the first encountered.

The number of selected covariates can be large (Fig. 1, available as supplementary data at *Bioinformatics* online). We used the Least Absolute Shrinkage and Selection Operator (LASSO) to select covariates from the identified adjustment sets.

The estimated coefficients $\hat{\beta }$ minimize the following optimization objective:


$$\frac{1}{2n}\sum_{i=1}^{n}{\left(y_{i}-X_{i}^{⊤}\beta \right)}^{2}+\lambda \sum_{j=1}^{p}|\beta_{j}|,$$

where $y_{i}$ is the outcome (side effect), $X_{i}$ is the vector of predictors (covarites in an adjustment set) for observation *i*, λ≥0 is a regularization parameter, and *p* is the number of covariates in the adjustment set. Covariates with non-zero coefficients in $\hat{\beta }$ are considered selected. This leads to smaller, more interpretable sets of covariates (Urminsky *et al.* 2016, Ye *et al.* 2021). Following the methodology in (Urminsky *et al.* 2016), we fitted two separate LASSO models—one for the indication and one for the side effect—and used the union of the variables selected in either model for adjustment. To optimize the models, we selected the regularization parameter (λ) that minimized the 10-fold cross-validation error. As depicted in Fig. 3, available as supplementary data at *Bioinformatics* online, LASSO shrinks the number of selected covariates.

## 3 Results

### 3.1 A framework for knowledge-based causal mediation analysis

We developed a method to identify post-marketing adverse effects of drugs from large-scale observational cohorts while controlling for confounding. We model this task as a mediation problem: testing whether a drug mediates the effect between an indication and a side effect. We then apply causal mediation analysis to identify drugs that significantly mediate associations between diseases.

To generate plausible hypotheses and control for confounding at scale, we first constructed a Causal Knowledge Graph (CKG) (Section 2.2). We define a CKG as a structure consisting of a knowledge graph, a subset of relations marked as “causal,” a probability space, a mapping between knowledge graph nodes and events in the probability space, and a set of constraints that ensure that the relational semantics in the knowledge graph is reflected in the probability space. We build a Drug–Disease Causal Knowledge Graph (DD-CKG) that enables us to identify mediating effects of drugs. The DD-CKG integrates disease progression, drug indications, side effects of drugs, and hierarchical relations from the ICD-10 hierarchy mapped as described in Supplementary section Mapping of medications and conditions, available as supplementary data at *Bioinformatics* online. We consider edges representing disease progression and the inverse of indication edges (i.e. that a disease diagnoses may lead to the prescription of the drug) as causal. We empirically assign a probability distribution to the CKG based on longitudinal cohort data in UK Biobank (Section 2.1).

The DD-CKG allows us to automate three key steps in mediation analysis: (i) posing hypotheses consistent with background knowledge, (ii) identifying confounding structures, and (iii) constraining the probability distribution to respect the KG’s prior semantics. We focus on pairs of diseases $D_{1}$ and $D_{2}$ that satisfy the following conditions: (a) a drug *M* is prescribed for $D_{1}$; (b) *M* is not indicated for both $D_{1}$ and $D_{2}$; and (c) *M* does not list both $D_{1}$ and $D_{2}$ as side effects (Fig. 1C). Because there are many disease pairs, we focus on two sets of disease pairs that may indicate disease progression: (1) the disease pairs that are explicitly linked in DD-CKG with a disease progression edge (causal set, 12 561 disease–disease pairs), and (2) diseases that are significantly co-morbid in UK Biobank and where one disease more often occurs before the other disease (comorbidity set, 81 610 disease–disease pairs).

### 3.2 Mediation analysis reveals highly concordant side effects

To apply causal mediation analysis, we used two observational cohorts, UK Biobank (UKB) and MIMIC-IV (Supplementary section Sample selection, available as supplementary data at *Bioinformatics* online). We used these cohorts to assign a probability distribution to the DD-CKG, and applied logical constraints from the DD-CKG to this distribution to make it consistent with the semantics of subsumption relations (Section 2.2). To adjust for confounding, we considered both demographic and socioeconomic factors, as well as two additional adjustment criteria: the backdoor and disjunctive cause criteria, applied to the DD-CKG (see Section 2.5). For each hypothesis, we computed the Average Causal Mediation Effect (ACME) (Tingley *et al.* 2014) of the drug, adjusting for the identified confounders. This analysis allows us to assess whether the drug significantly mediates the relationship between indications and possible side effect, indicating a direct effect of the drug on the outcome.

We computed the ACME of both sets of disease—disease pairs (the comorbidity set and the causal set), using different methods to adjust for confounding. We find that selecting confounders using the disjunctive cause criterion followed by selection through LASSO resulted in most testable hypotheses on the causal set, and due to the larger size of the comorbidity set, we only applied this confounder control on the comorbidity set. A positive ACME indicates that the drug may contribute to the occurrence of the outcome disease, while a negative ACME indicates that the drug may reduce the likelihood of the outcome disease. We focus only on positive ACME and compared resulting drug–disease pairs to existing adverse event databases. Because the used cohorts only share a limited number of hypotheses with sufficient sample sizes, we evaluated each cohort separately. Table 1 shows the results of the ACME and the comparison to existing databases (see Supplementary section Side effect evaluation, available as supplementary data at *Bioinformatics* online).

**Table 1.** Summary of mediation analysis results by hypothesis set and adjustment method.^a^

Hypotheses set	Adjustment method	N/A	Insig.	+ ACME	− **ACME**	Precision	Recall	F1
Causal	Backdoor adjustment	8459	2433	1200	1578	0.876	0.084	0.153
Causal	LASSO of backdoor adjustment	3226	4908	3237	2299	0.905	0.233	0.371
Causal	Disjunctive cause	10 694	1269	860	847	0.859	0.059	0.110
Causal	LASSO of disjunctive cause	2128	5287	3910	2345	0.907	0.282	0.431
Comorbidity	LASSO of disjunctive cause	34 643	20 802	30 724	13 158	0.749	0.282	0.410

a N/A indicates hypotheses that could not be tested; Insig denotes non-significant *P*-values; **+/**− ACME represents significant Average Causal Mediation Effects (ACMEs). We used Benjamini–Hochberg correction for all tests.

Additionally, we tested the performance of our predictions against a custom expert-curated set of adverse effects of drugs (Ryan *et al.* 2013). The set contains 165 positively and negatively annotated drug-outcome pairs of which we only tested the ones that coincide with our hypotheses set (*n* = 56). We found that the LASSO of the disjunctive cause applied on the comorbidity set achieved a precision of 70.0% and a recall of 60.6% when evaluated on the overlapping drug and outcome pairs. However, we were unable to test the other set of hypotheses due to the limited size of the testing set.

We find that the mediation analysis over the DD-CKG can reveal both known and novel drug effects. Using edges that are included in the DD-CKG as candidates, we find that most identified effects are already known and contained in a drug effect database (precision up to 0.907). This is expected as the pairs included in the DD-CKG are supported by literature and therefore likely correspond to established effects. Using significantly comorbid diseases in UKB, on the other hand, revealed more candidate drug effects (30 724 significant positive effects), the same recall, but lower precision (i.e. more potentially novel effects).

To further evaluate the drug effects we identify from the comorbidity set, we used them to compute side-effect similarity between drugs and tested whether drugs with higher side effect similarity share indications (see Supplementary section Prediction of shared indications and Fig. 1, available as supplementary data at *Bioinformatics* online). While using only drug effects predicted by the causal mediation analysis yields a lower performance (ROCAUC: 0.604) than using drug effects from the OnSIDES database (ROCAUC: 0.620), combining OnSIDES and our predicted drug effects significantly improves the prediction of shared indications(ROCAUC: 0.632, p≪0.001, Mann Whitney U test). Additionally, the associated Precision-Recall curve shows that the causal mediation analysis results yield higher area under the curve than OnSIDES (Fig. 2, available as supplementary data at *Bioinformatics* online).

## 4 Discussion

### 4.1 Mediation analysis explain disease–disease relationships

Analysis of the comorbidity set revealed drug-mediated disease relationships. *Vincristine* fully mediated the effect of *Non-Hodgkin lymphoma* on *Tumor Lysis Syndrome* (ACME = 0.001, proportion mediated = -0.905; see Fig. 3, available as supplementary data at *Bioinformatics* online). The negative proportion indicates a suppression effect (MacKinnon *et al.* 2000), consistent with Tumor Lysis Syndrome occurring primarily as a chemotherapy complication (Williams and Killeen 2019).

*Simvastatin* mediated the link between *Mixed hyperlipidemia* and *COPD* (ACME = 0.04177, proportion mediated = −0.377; Fig. 4, available as supplementary data at *Bioinformatics* online). While statins rarely cause interstitial lung disease, COPD has not been reported (Fernández *et al.* 2008, Huang *et al.* 2013). Since smoking (Laniado-Laborín 2009) was controlled for in UKB data, this suggests potential simvastatin-mediated effects.

Expert curation identified additional unexpected associations. Proton pump inhibitors (lansoprazole, omeprazole) mediated GERD-myocardial infarction links (Strand *et al.* 2017). ACE inhibitors (lisinopril, enalapril, perindopril, ramipril) mediated nephropathy-myocardial infarction associations (Izzo Jr and Weir 2011), suggesting class-wide effects despite ACEs’ widespread use in nephropathy (Bhandari *et al.* 2022) and reported myocardial infarction risks (Na Takuathung *et al.* 2022). While PPI-associated myocardial infarction risk is absent from drug labels (Elias and Targownik 2019) and recent ambulatory data analyses (Ma *et al.* 2020), it was previously identified in Stanford EHR data mining of 1.8 million individuals (Shah *et al.* 2015, Ariel and Cooke 2019).

### 4.2 Disease severity as a confounder

We used comorbidity count and medication count as proxies for disease severity (unobservable in our dataset). Among 900 manually evaluated false positives, 6% likely represented disease progression rather than drug mediation. This unobserved confounding occurs when drug *M* is prescribed for an unmitigated condition that progresses severely; in milder responsive cases, no outcome disease appears, making *M* falsely appear to mediate $D_{2}$.

For example, *Disorder of kidney and ureter* (N28.9) associated with *Malignant renal neoplasm* (C64) through eleven hypertension drugs. These drugs do not cause malignancy, but hypertension, dialysis, and renal failure are established malignancy risk factors (Chow *et al.* 2010), suggesting severity-driven associations. Similarly, progressions like *Mixed hyperlipidemias*, *Hyperlipidemia*, *Hyperglycemia*, and *Alcohol dependence* to *Fatty liver* (Israelsen *et al.* 2024) were incorrectly flagged as drug-mediated.

While weighted severity indices like the Charlson comorbidity index (Charlson *et al.* 1987) provide more refined clinical assessments of severity, they are designed primarily for mortality prediction and require condition-specific weighting. Our approach treats all conditions equally for confounding adjustment, which is conservative but may not fully capture differences in disease severity. Future work could explore incorporating validated severity indices tailored to specific clinical contexts.

### 4.3 Causal knowledge graphs

A major contribution of our work is the development of a novel class of Causal Knowledge Graphs (CKGs), which extend traditional knowledge graphs with formal semantics that enable causal inference while preserving relational semantics, support deconfounding via explicitly marked causal edges, and facilitate causal hypothesis formulation directly aligned with encoded background knowledge. Prior work has combined knowledge graphs with causal inference through different approaches. Some methods consider knowledge graphs where relations have causal interpretations, with the graphical model coinciding entirely or partially with the knowledge graph (Gopalakrishnan *et al.* 2023, Tan *et al.* 2024). These focus on inferring causal relations using relational semantics but do not integrate the graph with the causal model’s probability distribution. CauseKG (Huang and Vidal 2024) integrates the probability distribution with relational semantics, but is limited to “identity” between nodes and sub-property considerations.

Our framework combines two distinct types of knowledge for causal inference. Knowledge graphs encode qualitative relationships representing connections between entities that are documented in scientific literature or clinical knowledge bases, while empirical probability distributions from longitudinal cohorts quantify the strength of these relationships in real populations. The knowledge graph defines the causal structure and enables the identification of confounders based on established biological mechanisms; the longitudinal cohort data enables hypothesis testing and effect estimation. Different data sources naturally vary in scope and coverage, reflecting how biomedical knowledge is currently organized. This creates two challenges: reliability (incorrect or weakly supported edges) and completeness (missing true relationships). Our approach addresses these through distinct mechanisms. For reliability, our validation approach uses independent empirical evidence from large-scale cohorts rather than relying solely on graph structure, effectively minimizing the effect of false knowledge. For completeness, our framework enables future enrichment through deductive reasoning and knowledge graph completion methods, as we detail below.

On the other hand, our approach establishes a link between knowledge graph semantics and a probability space, enabling a deep integration between knowledge graph semantics and the causal model’s probability distribution. Specifically, we show that this integration allows exploitation of subsumption relations between diseases, i.e. deductive subsumption reasoning that constrains the probability distribution. While we have only shown this form of reasoning, CKGs can also enable other forms of deductive inference. Crucial for other types of inference is that we do not identify the knowledge graph with the causal graph, but rather make the causal structure a subset of the knowledge graph. This allows the use of the complete knowledge graph (including both causal and non-causal relations) combined with rules to deductively infer edges to add to the knowledge graph, and then generate an enriched causal graph from both asserted and inferred edges. Moreover, the relation between the knowledge graph and causal structure in CKGs could also be combined with knowledge graph completion methods (Chen *et al.* 2020) to further enrich both the knowledge graph and causal structure potentially overcoming the limitation of missing edges. This way, missing knowledge could potentially be recovered In the future, approaches that combine causal semantics with the formal semantics of more expressive knowledge representation languages, such as the Web Ontology Language (OWL) (Consortium, W. W. W *et al.* 2012), may be explored.

### 4.4 Application to longitudinal cohorts and the electronic health record

The causal mediation framework we propose is general and can be applied to other longitudinal cohorts. The application of our framework requires longitudinal data including diagnoses and drug prescriptions, and the ability to map the diagnoses and prescriptions to standardized identifiers that are included in our knowledge graph. This standardization is a limiting factor especially in biobank-style cohorts where information from multiple different sources (e.g. primary care, hospital records, and self-reported information from surveys) is integrated. We standardized medications using a combination of a lexical approach and a Large Language Model (LLM). Therefore, we acknowledge that incorrect mappings may exist in our results. Nonetheless, our mapping approach can potentially be extended in the future by considering other LLMs or other approaches to map labels to a structured vocabulary.

In our work, drug incidence was based on self-reported medication use in UKB, which lacks accurate information on prescription timing and dosage. More accurate information could be obtained from the Electronic Health Record (EHR). However, systematically evaluating our results against existing curated datasets proved challenging. Our aim is to detect novel side effects from observed disease-disease relationships rather than to validate known adverse events. Therefore, we are testing whether we can establish causal relationships using our method, not whether existing annotations hold empirically. This challenge is illustrated in the evaluation against the small curated dataset by (Ryan *et al.* 2013) in the results section where we were able to achieve high precision but struggled to evaluate against the other set of hypotheses due to the small overlap. This low overlap underscores that our approach explores a largely complementary space of potential drug effects beyond those already documented in curated resources.

Furthermore, in our CKG, we used ICD-coded diagnoses to represent observed phenotypes. While these ensure clinician validation, transient or mild side effects are not observed. To include this information, electronic health records could be mined for milder effects using text mining approaches Slater *et al.* (2021); Iyer *et al.* (2014), and using vocabularies that can capture these effects.

## 5 Conclusion

We have developed a novel approach to identify rare adverse drug events from longitudinal health data. Our approach is enabled by a novel framework that combines knowledge graphs with causal models, which we call Causal Knowledge Graphs (CKGs). CKGs enable the identification and control of confounding variables, allow entailment of causal relations using deductive inference, and can constrain a probability distribution with background domain knowledge. These properties of CKGs together with the availability of large amounts of biomedical domain knowledge in the form of biomedical knowledge graphs, as well as large longitudinal cohorts, allows us to find rare adverse events with low effect size that have been missed in other studies. Moreover, our analysis relies on standard identifiers used in health records and standard representation formats for biomedical knowledge; it therefore has the potential to be extended to other applications of causal inference where structured domain knowledge and observational data are both available, and the observations can be linked to entities in the domain knowledge.

## Supplementary Material

btaf661_Supplementary_Data

## Data Availability

Data is available through https://github.com/bio-ontology-research-group/Mediation-Analysis-using-Causal-Knowledge-Graph.

## References

[btaf661-B1] Alterovitz G , Ramoni M. Knowledge-Based Bioinformatics: From Analysis to Interpretation. John Wiley & Sons, Hoboken, New Jersey, USA, 2011.

[btaf661-B2] Ariel H , Cooke JP. Cardiovascular risk of proton pump inhibitors. Methodist Debakey Cardiovasc J 2019;15:214–9. 31687101 10.14797/mdcj-15-3-214 PMC6822659

[btaf661-B3] Bhandari S , Mehta S, Khwaja A et al; STOP ACEi Trial Investigators. Renin–angiotensin system inhibition in advanced chronic kidney disease. N Engl J Med 2022;387:2021–32. 36326117 10.1056/NEJMoa2210639

[btaf661-B4] Charlson ME , Pompei P, Ales KL et al A new method of classifying prognostic comorbidity in longitudinal studies: development and validation. J Chronic Dis 1987;40:373–83. 3558716 10.1016/0021-9681(87)90171-8

[btaf661-B5] Chen Z , Wang Y, Zhao B et al Knowledge graph completion: a review. IEEE Access 2020;8:192435–56.

[btaf661-B6] Chow W-H , Dong LM, Devesa SS. Epidemiology and risk factors for kidney cancer. Nat Rev Urol 2010;7:245–57. 20448658 10.1038/nrurol.2010.46 PMC3012455

[btaf661-B8] Elias E , Targownik LE. The clinician’s guide to proton pump inhibitor related adverse events. Drugs 2019;79:715–31. 30972661 10.1007/s40265-019-01110-3

[btaf661-B9] Fernández AB , Karas RH, Alsheikh-Ali AA et al Statins and interstitial lung disease. Chest 2008;134:824–30. 18689579 10.1378/chest.08-0943

[btaf661-B10] Forslund T , Carlsson AC, Ljunggren G et al Patterns of multimorbidity and pharmacotherapy: a total population cross-sectional study. Fam Pract 2021;38:132–40. 32766818 10.1093/fampra/cmaa056 PMC8006765

[btaf661-B11] Fukuto K , Takagi T, Tian Y-S. Predicting the side effects of drugs using matrix factorization on spontaneous reporting database. Sci Rep 2021;11:23942. 34907245 10.1038/s41598-021-03348-y PMC8671428

[btaf661-B12] Gentreau M , Rukh G, Miguet M et al The effects of statins on cognitive performance are mediated by low-density lipoprotein, C-reactive protein, and blood glucose concentrations. J Gerontol A Biol Sci Med Sci 2023;78:1964–72. 37431946 10.1093/gerona/glad163 PMC10613010

[btaf661-B13] Gopalakrishnan S , Chen VZ, Dou W et al Text to causal knowledge graph: a framework to synthesize knowledge from unstructured business texts into causal graphs. Information 2023;14:367.

[btaf661-B14] Hammad TA , Afsar S, McAvoy LB et al Aspects to consider in causality assessment of safety signals: broadening the thought process. Front Drug Saf Regul 2023;3:1193413. 40980101 10.3389/fdsfr.2023.1193413 PMC12443079

[btaf661-B15] Hidalgo CA , Blumm N, Barabási A-L et al A dynamic network approach for the study of human phenotypes. PLoS Comput Biol 2009;5:e1000353. 19360091 10.1371/journal.pcbi.1000353 PMC2661364

[btaf661-B16] Hogan A , Blomqvist E, Cochez M et al Knowledge graphs. ACM Comput Surv 2022;54:1–37.

[btaf661-B17] Hu W , Li H, Zeng L et al Data mining in faers: association of newer-generation h1-antihistamines with nervous system disorders. BMC Pharmacol Toxicol 2024;25:95–11. 39696617 10.1186/s40360-024-00822-x PMC11656970

[btaf661-B18] Huang H , Vidal M-E. Causekg: a framework enhancing causal inference with implicit knowledge deduced from knowledge graphs. IEEE Access 2024;12:61810–27.

[btaf661-B19] Huang L-K , Tsai M-J, Tsai H-C et al Statin-induced lung injury: diagnostic clue and outcome. Postgrad Med J 2013;89:14–9. 23043128 10.1136/postgradmedj-2011-130209 PMC3533381

[btaf661-B20] Imai K , Keele L, Tingley D. A general approach to causal mediation analysis. Psychol Methods 2010;15:309–34. 20954780 10.1037/a0020761

[btaf661-B21] Israelsen M , Francque S, Tsochatzis EA et al Steatotic liver disease. Lancet 2024;404:1761–78. 39488409 10.1016/S0140-6736(24)01811-7

[btaf661-B22] Iyer SV , Harpaz R, LePendu P et al Mining clinical text for signals of adverse drug–drug interactions. J Am Med Inform Assoc 2014;21:353–62. 24158091 10.1136/amiajnl-2013-001612 PMC3932451

[btaf661-B23] Izzo JL Jr , Weir MR. Angiotensin-converting enzyme inhibitors: angiotensin-converting enzyme inhibitors. J Clin Hypertens (Greenwich) 2011;13:667–75. 21896148 10.1111/j.1751-7176.2011.00508.x PMC8108813

[btaf661-B24] Laniado-Laborín R. Smoking and chronic obstructive pulmonary disease (COPD). Parallel epidemics of the 21st century. Int J Environ Res Public Health 2009;6:209–24. 19440278 10.3390/ijerph6010209 PMC2672326

[btaf661-B25] Ma C , Shaheen AA, Congly SE et al Interpreting reported risks associated with use of proton pump inhibitors: residual confounding in a 10-year analysis of national ambulatory data. Gastroenterology 2020;158:780–2.e3. 31678304 10.1053/j.gastro.2019.10.023

[btaf661-B26] MacKinnon DP , Krull JL, Lockwood CM. Equivalence of the mediation, confounding and suppression effect. Prev Sci 2000;1:173–81. 11523746 10.1023/a:1026595011371 PMC2819361

[btaf661-B27] Mozzicato P. Meddra: an overview of the medical dictionary for regulatory activities. Pharm Med 2009;23:65–75.

[btaf661-B28] Na Takuathung M , Sakuludomkan W, Khatsri R et al Adverse effects of angiotensin-converting enzyme inhibitors in humans: a systematic review and meta-analysis of 378 randomized controlled trials. Int J Environ Res Public Health 2022;19:8373. 35886227 10.3390/ijerph19148373 PMC9324875

[btaf661-B29] Nelson SJ , Zeng K, Kilbourne J et al Normalized names for clinical drugs: Rxnorm at 6 years. J Am Med Inform Assoc 2011;18:441–8. 21515544 10.1136/amiajnl-2011-000116 PMC3128404

[btaf661-B30] Pande S. Causality or relatedness assessment in adverse drug reaction and its relevance in dermatology. Indian J Dermatol 2018;63:18–21. 29527021 10.4103/ijd.IJD_579_17 PMC5838749

[btaf661-B31] Pearl J. Causality. 2nd edn. Cambridge, UK: Cambridge University Press, 2009a.

[btaf661-B32] Pearl J. Causality and Structural Models in Social Science and Economics. Cambridge, UK: Cambridge University Press, 2009b, 133–72.

[btaf661-B33] Pearl J. Interpretation and identification of causal mediation. Psychol Methods 2014;19:459–81. 24885338 10.1037/a0036434

[btaf661-B34] Pham T , Tao X, Zhang J et al Constructing a knowledge-based heterogeneous information graph for medical health status classification. Health Inf Sci Syst 2020;8:10. 32117570 10.1007/s13755-020-0100-6 PMC7021844

[btaf661-B35] Prud’hommeaux E , Seaborne A. SPARQL Query Language for RDF. W3C Recommendation. 2008.

[btaf661-B36] Raj N , Fernandes S, Charyulu NR et al Postmarket surveillance: a review on key aspects and measures on the effective functioning in the context of the United Kingdom and Canada. Ther Adv Drug Saf 2019;10:2042098619865413. 31384423 10.1177/2042098619865413 PMC6661791

[btaf661-B37] Ryan PB , Schuemie MJ, Welebob E et al Defining a reference set to support methodological research in drug safety. Drug Saf 2013;36:S33–47. 24166222 10.1007/s40264-013-0097-8

[btaf661-B38] Shah NH , LePendu P, Bauer-Mehren A et al Proton pump inhibitor usage and the risk of myocardial infarction in the general population. PLoS One 2015;10:e0124653. 26061035 10.1371/journal.pone.0124653 PMC4462578

[btaf661-B39] Slater LT , Bradlow W, Ball S et al Improved characterisation of clinical text through ontology-based vocabulary expansion. J Biomed Semantics 2021;12:7. 33845909 10.1186/s13326-021-00241-5 PMC8042947

[btaf661-B40] Steindel SJ. International classification of diseases, clinical modification and procedure coding system: descriptive overview of the next generation HIPAA code sets. J Am Med Inform Assoc 2010;17:274–82. 20442144 10.1136/jamia.2009.001230 PMC2995704

[btaf661-B41] Strand DS , Kim D, Peura DA. 25 years of proton pump inhibitors: a comprehensive review. Gut Liver 2017;11:27–37. 27840364 10.5009/gnl15502 PMC5221858

[btaf661-B42] Sudlow C , Gallacher J, Allen N et al Uk biobank: an open access resource for identifying the causes of a wide range of complex diseases of middle and old age. PLoS Med 2015;12:e1001779. 25826379 10.1371/journal.pmed.1001779 PMC4380465

[btaf661-B43] Tan FA , Desai J, Sengamedu SH. 2024. Enhancing fact verification with causal knowledge graphs and transformer-based retrieval for deductive reasoning. In: Schlichtkrull M, Chen Y, Whitehouse C et al (eds.), Proceedings of the Seventh Fact Extraction and VERification Workshop (FEVER). Miami, Florida, USA: Association for Computational Linguistics, 151–69.

[btaf661-B44] Tanaka Y , Chen HY, Belloni P et al OnSIDES database: Extracting adverse drug events from drug labels using natural language processing models. Med 2025. 10.1016/j.medj.2025.100642 PMC12256195 40179876

[btaf661-B45] Tatonetti NP , Ye PP, Daneshjou R et al Data-driven prediction of drug effects and interactions. Sci Transl Med 2012;4:125ra31. 10.1126/scitranslmed.3003377 PMC3382018 22422992

[btaf661-B46] Tchetgen E , Phiri K. Evaluation of medication-mediated effects in pharmacoepidemiology. *Epidemiology (Cambridge*, *Mass.)*, 2016;28:439–45. 10.1097/EDE.0000000000000610 PMC5389597 27984423

[btaf661-B47] Tennant PWG , Murray EJ, Arnold KF et al Use of directed acyclic graphs (DAGS) to identify confounders in applied health research: review and recommendations. Int J Epidemiol 2021;50:620–32. 33330936 10.1093/ije/dyaa213 PMC8128477

[btaf661-B48] Textor J , Van der Zander B et al Robust causal inference using directed acyclic graphs: the r package ‘dagitty’. Int J Epidemiol 2016;45:1887–94. 28089956 10.1093/ije/dyw341

[btaf661-B49] Tingley D , Yamamoto T, Hirose K et al mediation: R package for causal mediation analysis. J Stat Soft 2014;59:1–38.

[btaf661-B50] Toonsi S , Gauran II, Ombao H et al Causal relationships between diseases mined from the literature improve the use of polygenic risk scores. Bioinformatics 2024;40:btae639. 39460944 10.1093/bioinformatics/btae639 PMC11639291

[btaf661-B51] Trifirò G , Crisafulli S. A new era of pharmacovigilance: future challenges and opportunities. Front Drug Saf Regul 2022;2:866898.

[btaf661-B52] Urminsky O , Hansen C, Chernozhukov V. Using double-lasso regression for principled variable selection. SSRN J 2016. 10.2139/ssrn.2733374

[btaf661-B53] Vander Weele TJ , Shpitser I. A new criterion for confounder selection. Biometrics 2011;67:1406–13. 21627630 10.1111/j.1541-0420.2011.01619.x PMC3166439

[btaf661-B7] W3C OWL Working Group. OWL 2 Web Ontology Language Document Overview (Second Edition). W3C Recommendation, 2012. http://www.w3.org/TR/2012/REC-owl2-overview-20121211/.

[btaf661-B54] Williams SM , Killeen AA. Tumor lysis syndrome. Arch Pathol Lab Med 2019;143:386–93. 30499695 10.5858/arpa.2017-0278-RS

[btaf661-B55] Xu T , Brandmaier S, Messias AC et al Effects of metformin on metabolite profiles and LDL cholesterol in patients with type 2 diabetes. Diabetes Care 2015;38:1858–67. 26251408 10.2337/dc15-0658

[btaf661-B56] Ye Z , Zhu Y, Coffman DL. Variable selection for causal mediation analysis using lasso-based methods. Stat Methods Med Res 2021;30:1413–27. 33755518 10.1177/0962280221997505 PMC8189011

[btaf661-B57] Zhan B. Application and investigation of knowledge graph in biomedical field. ACE 2024;88:86–92.

[btaf661-B58] Zhang W , Peissig P, Kuang Z et al Adverse drug reaction discovery from electronic health records with deep neural networks. Proc ACM Conf Health Inference Learn 2020;2020:30–9. 10.1145/3368555.3384459 PMC7718770 33283213

[btaf661-B59] Zheng NS , Kerchberger VE, Borza VA et al An updated, computable medication-indication resource for biomedical research. Sci Rep 2021;11:18953. 34556781 10.1038/s41598-021-98579-4 PMC8460636

