# Supplementary material for: Causal knowledge graph analysis identifies adverse drug effects
Source: Bioinformatics. 2025 Dec 12;42(1):btaf661. doi: 10.1093/bioinformatics/btaf661 (PMC12790815; doi:10.1093/bioinformatics/btaf661)
Supplement: btaf661_Supplementary_Data [file btaf661_supplementary_data.pdf]

Supplementary materials for: Causal knowledge graph analysis identifies adverse drug effects

RxNorm ID	Drug name	Candidate drugs	Prompt
358263	Tadalafil	“Adcirca (tadalafil)” and “Tadalafil (Cialis)”	If the medication “Tadalafil” is commonly expressed as any of the medications in the following list, answer only with the number/s of the entry/entries separated by commas, otherwise, answer “no”.<br>1- Adcirca (Tadalafil)<br>2- Tadalafil (Cialis)

Table S1: Example prompt for matching candidate drug names

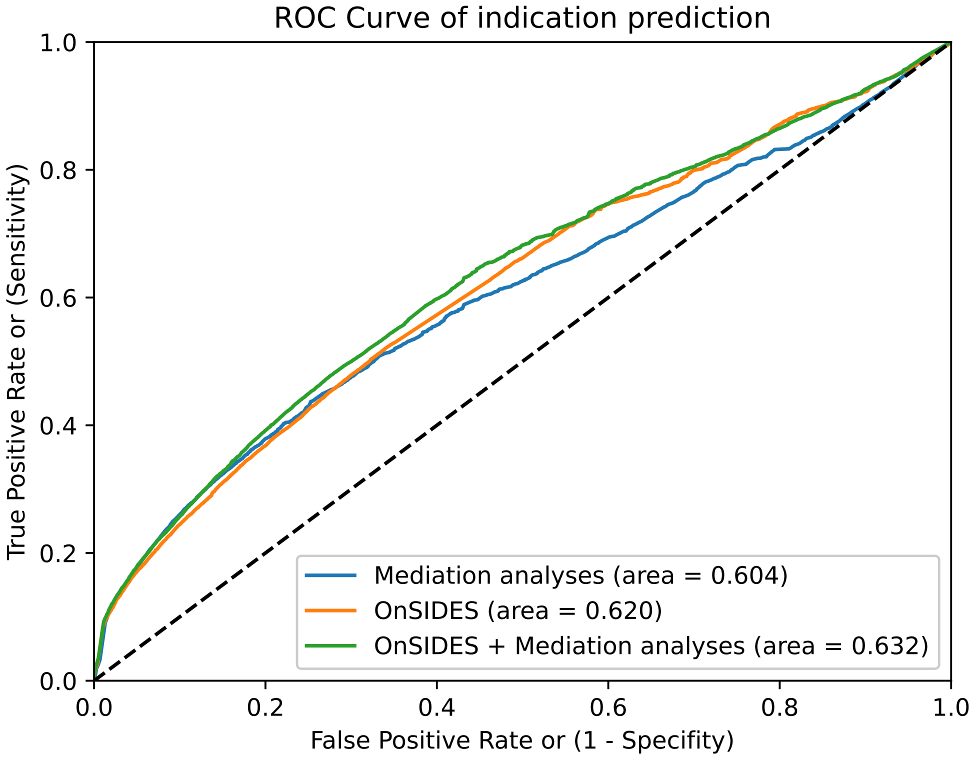

Figure S1: ROC AUC for predicting shared drug indications based on side effect similarity scores.

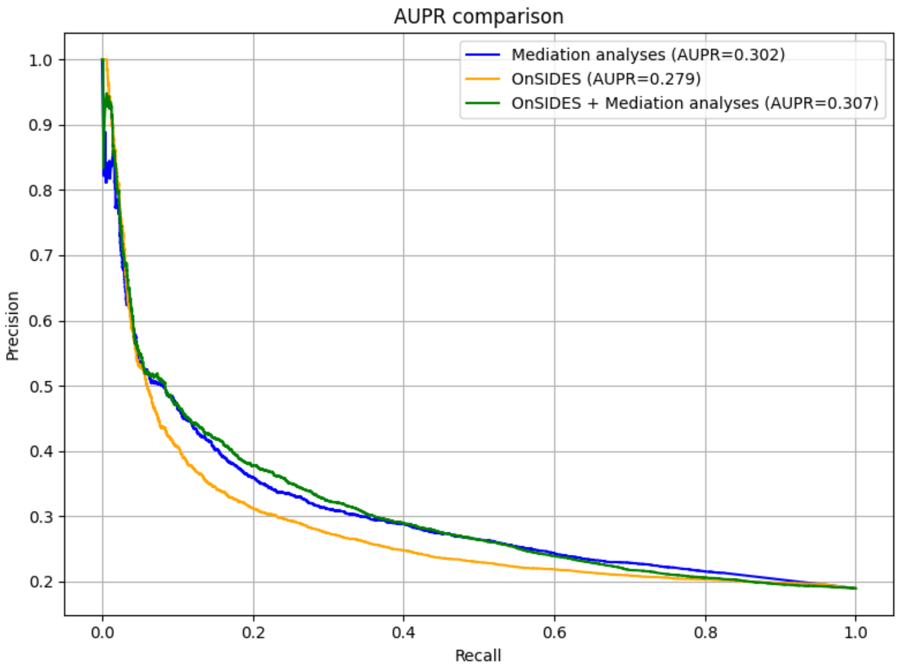

Figure S2: Precision-Recall curve for predicting shared drug indications based on side effect similarity scores.

Mapping of medications and conditions

Because UKB and MIMIC-IV use different identifiers for diagnoses and drugs, they are not directly mappable. We developed a hybrid approach to map drugs from RxNorm to both cohorts. For each RxNorm drug, we first attempted to find an exact lexical match between its preferred name and drug names recorded in UKB and MIMIC-IV. If no exact match was available, we identified as candidates any medications in UKB or MIMIC-IV that partially matched the RxNorm name on a word-by-word basis. To resolve these partial matches, we used the `Llama-3-70B-Instruct` (Grattafiori *et al.*, 2024) Large Language Model (LLM). For each RxNorm drug, we prompted the LLM (see Table S1) to select the most appropriate candidates from the list.

To evaluate the accuracy of the LLM-based mapping, we randomly selected 50 drugs (25 from UKB and 25 from MIMIC-IV) for manual review. An expert (P.N.S) examined the candidate lists for each RxNorm drug, using the preferred name as reference. The expert determined that a candidate was a correct match if the RxNorm drug — based on its preferred name — was fully or partially represented in the candidate, meaning the candidate correctly corresponded to the intended drug entity, even if the names were not identical. Based on this manual curation, the LLM achieved a precision of 0.70, a recall of 0.83, and an F1-score of 0.76. Using this hybrid approach, we mapped 941 drugs from RxNorm to UKB, of which 233 were based on exact match. We also mapped 839 drugs from RxNorm to MIMIC-IV of which 541 were based on an exact match. The manually curated drug mappings and the mapping results of the LLM are available in the Github repository.

Both OnSIDES and OFFSIDES report side effects using the Medical Dictionary of Drug Regulatory Activities (MedDRA). On the other hand, the causal relations between diseases in the graph we use are reported using the International Classification of Diseases, tenth revision, Clinical Modification (ICD-10-CM) codes. Therefore, we mapped conditions from MedDRA to ICD-10-CM using the Unified Medical Language System (UMLS) (Bodenreider, 2004). Furthermore, we used UMLS to map ICD-9 codes to ICD-10 to represent diagnoses.

Sample selection

To confirm the incidence of indications and side effects, we used the reported codes of ICD-10 diagnoses and their dates for each individual in the UKB (Data-Field 41270). For MIMIC-IV, we retrieved all ICD-10 and ICD-9 codes with the reported dates for each individual. In the UKB, information about drug prescriptions includes self-reported information where individuals were asked to provide information about medications they were taking (Data-Field 20003). The UKB provides data on multiple visits of individuals to their centers and prescriptions were reported at each visit to the assessment centers. In the MIMIC-IV dataset, we used pharmacy records of prescribed drugs and their duration.

To obtain longitudinal data, we first excluded individuals without follow-up data. We then excluded samples where the variables did not follow the required temporal order: indication diagnosis, followed by drug use, and then side effect diagnosis. Specifically, we excluded individuals diagnosed with the outcome before either the drug or indication. Since drugs can be

taken intermittently or consecutively, we excluded those who reported using the drug only before the indication diagnosis.

Prediction of shared indications

As intially proposed in (Campillos *et al.*, 2008), side effect similarity can be used to predict if two drugs share indications. Following the approach in (Tatonetti *et al.*, 2012; Tanaka *et al.*, 2024), we repeated the analysis by computing the pairwise Tanimoto coefficient score for drugs based on their side effects. That is, for two drugs A and B and their corresponding sets of side effects SE_A and SE_B, their similarity $Sim(A, B)$ is calculated by:

$$Sim(A, B) = \frac{|SE_A \cap SE_B|}{|SE_A \cup SE_B|},$$

We compared different sources using side effects either reported by OnSIDES, obtained from our analysis, or the union of both. For each configuration, we computed pairwise drug similarity scores using the Tanimoto coefficient and applied z-score normalization to the resulting similarity matrix. To evaluate whether these scores could predict whether two drugs share a common indication (a binary outcome), we assessed the predictive performance using the area under the receiver operating characteristic curve (ROC AUC).

Side effect evaluation

We used the OnSIDES and OFFSIDES datasets to construct a reference set of known drug-–side effect pairs. For each drug–side effect pair identified by our mediation analysis, we classified it as a true positive if it appeared in the reference set, and as a false positive if it did not. False negatives were defined as drug–side effect hypotheses that (i) were generated in Section *Generation of hypotheses*, (ii) appear in the reference set, but (iii) did not reach statistical significance in our analysis.

SPARQL query for contradicting hypotheses

```
PREFIX kg: <http://CKG.org/>

SELECT DISTINCT ?drug
WHERE {
  # 1. Find drugs indicated for disease A
  ?diseaseA kg:hasIndication ?drug .

  # 2. Exclude drugs also indicated for disease B
  FILTER NOT EXISTS {
    ?diseaseB kg:hasIndication ?drug .
  }

  # 3. Exclude drugs having both diseases as side effects
  FILTER NOT EXISTS {
    ?drug kg:hasSideEffect ?diseaseA .
    ?drug kg:hasSideEffect ?diseaseB .
  }
}
```

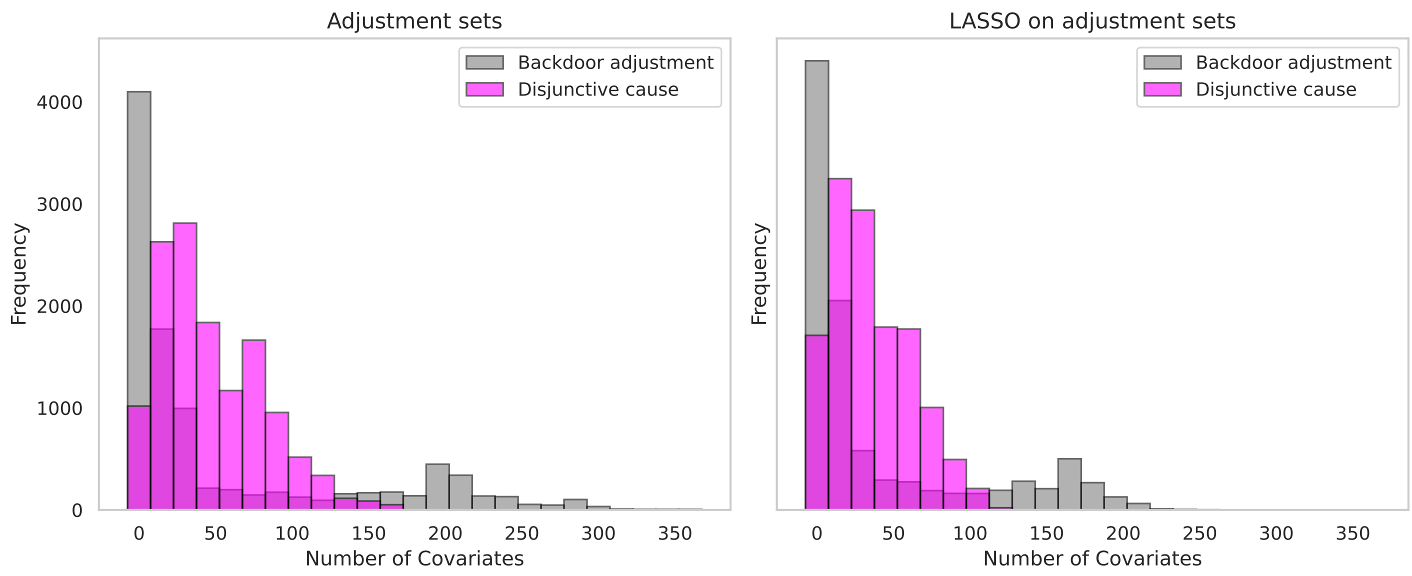

Figure S3: Distribution of the number of covariates per method.

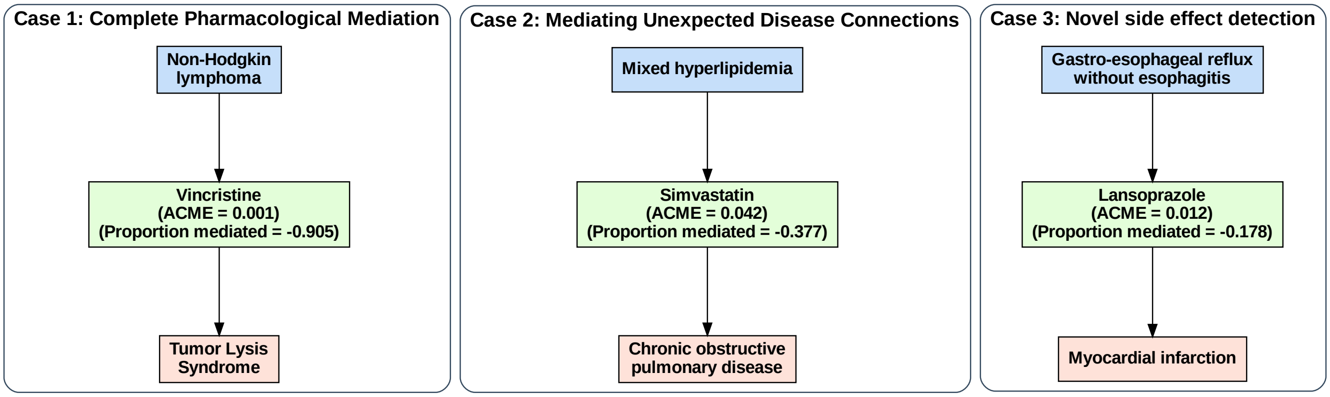

Figure S4: Mediation cases representing different explanations of inter–disease relationships.

References

Bodenreider, O. and Bodenreider, O. and Bodenreider, O. (2004). The unified medical language system (umls): integrating biomedical terminology. *Nucleic acids research*, **32**(suppl_1), D267–D270.

Campillos, M., Kuhn, M. *et al.* (2008). Drug target identification using side-effect similarity. *Science*, **321**(5886), 263–266.

Grattafiori, A., Dubey, A. *et al.* (2024). The llama 3 herd of models. *arXiv preprint arXiv:2407.21783*.

Tanaka, Y., Chen, H. Y. *et al.* (2024). Onsides (on-label side effects resource) database: extracting adverse drug events from drug labels using natural language processing models. *medRxiv*, pages 2024–03.

Tatonetti, N. P., Ye, P. P. *et al.* (2012). Data-driven prediction of drug effects and interactions. *Science translational medicine*, **4**(125), 125ra31–125ra31.
